# The Mystery of Diabetic Cardiomyopathy: From Early Concepts and Underlying Mechanisms to Novel Therapeutic Possibilities

**DOI:** 10.3390/ijms22115973

**Published:** 2021-06-01

**Authors:** Petra Grubić Rotkvić, Zrinka Planinić, Ana-Marija Liberati Pršo, Jozica Šikić, Edvard Galić, Luka Rotkvić

**Affiliations:** 1 Clinic of Internal Medicine, University Hospital “Sveti Duh”, Sveti Duh 64, 10000 Zagreb, Croatia; zrinkaplaninic@gmail.com (Z.P.); anamarijaliberati@gmail.com (A.-M.L.P.); josicas1@gmail.com (J.Š.); edvard.galic1@gmail.com (E.G.); 2 Faculty of Food Technology and Biotechnology, University of Zagreb, Pierottijeva 6, 10000 Zagreb, Croatia; 3 School of Medicine, University of Zagreb, Salata 2, 10000 Zagreb, Croatia; 4 Magdalena Clinic for Cardiovascular Diseases, Ljudevita Gaja 2, 49217 Krapinske Toplice, Croatia; luka.rotkvic@magdalena.hr

**Keywords:** diabetes mellitus, diabetic cardiomyopathy, heart failure

## Abstract

Diabetic patients are predisposed to diabetic cardiomyopathy, a specific form of cardiomyopathy which is characterized by the development of myocardial fibrosis, cardiomyocyte hypertrophy, and apoptosis that develops independently of concomitant macrovascular and microvascular diabetic complications. Its pathophysiology is multifactorial and poorly understood and no specific therapeutic guideline has yet been established. Diabetic cardiomyopathy is a challenging diagnosis, made after excluding other potential entities, treated with different pharmacotherapeutic agents targeting various pathophysiological pathways that need yet to be unraveled. It has great clinical importance as diabetes is a disease with pandemic proportions. This review focuses on the potential mechanisms contributing to this entity, diagnostic options, as well as on potential therapeutic interventions taking in consideration their clinical feasibility and limitations in everyday practice. Besides conventional therapies, we discuss novel therapeutic possibilities that have not yet been translated into clinical practice.

## 1. Introduction

Diabetes mellitus is one of the biggest global public health problems and one of the top ten causes of death in the world, with the prevalence that has increased in recent decades in most developing and developed regions [1,2,3]. It is a metabolic disorder with long-term microvascular and macrovascular complications. There are two types of this disease, both susceptible to long-term vascular complications: type 1, insulin-dependent with autoimmune mechanisms as triggers for the disease, which accounts for 5–10% of all cases, and type 2, non-insulin-dependent, characterized by insulin resistance, which accounts for the remaining 90% of all cases of diabetes. The macrovascular complications, comprising coronary heart disease, stroke, and peripheral vascular disease, known as atherosclerotic cardiovascular disease, are the major causes of mortality, morbidity, and healthcare costs in diabetes [4,5]. Interestingly, about seventy percent of type 2 diabetes mellitus at age ≥65 years die from cardiovascular disease [6]. Moreover, diabetic patients are predisposed to diabetic cardiomyopathy, a specific form of cardiomyopathy which is characterized by the development of myocardial fibrosis, cardiomyocyte hypertrophy, and apoptosis that develops independently of concomitant macrovascular and microvascular diabetic complications. Its pathophysiology is multifactorial and poorly understood despite its great clinical importance since observational studies have identified an approximately two to fivefold risk of heart failure in patients with diabetes compared to individuals without diabetes and since it is associated with poor outcomes [5,7,8]. Furthermore, recent major cardiovascular outcomes trials in diabetes have underlined that heart failure is a critical outcome in diabetic patients and proposed that glucose-lowering drugs could influence the development and progression of heart failure in diabetes [9]. To be more accurate, SGLT2 inhibitors as the drugs that stimulate urinary glucose excretion according to their pivotal outcome trials (EMPA-REG OUTCOME trial, CANVAS Program, DECLARE-TIMI 58) have considerably reduced mortality in type 2 diabetes, most likely not driven by a reduction in atherothrombotic events but through prevention of heart failure [10,11,12]. These results served as a reminder of the neglected entity of diabetic cardiomyopathy and heart failure in diabetes. Nevertheless, the mechanisms leading to diabetic cardiomyopathy remain undetermined, and consequently, no specific therapeutic guideline has yet been established. This review focuses on the potential mechanisms contributing to this entity, diagnostic options, as well as on potential therapeutic interventions.

## 2. Definition

Diabetic cardiomyopathy implies the existence of abnormal myocardial structure (characterized by the development of cardiomyocyte hypertrophy, apoptosis, and fibrosis) and abnormal myocardial performance without the concomitant presence of hypertension, coronary artery disease (CAD), and valvular heart disease, in patients with diabetes mellitus [13]. The pathophysiology underlying this entity is multifactorial and could obviously be present in diabetic patients with CAD, hypertension, or valvular pathologies making it more difficult to separately evaluate the contribution of this cardiomyopathy to overall cardiac abnormalities in diabetes [14].

## 3. History

Diabetic cardiomyopathy was first described in 1972 when Rubler et al. found adverse structural changes in postmortem pathological findings from four diabetic patients without evidence of coronary artery or valve disease and was further confirmed in a 1974 Framingham Heart Study [8,15]. Despite considerable attention, with basic and clinical investigations in past decades, the concept of this clinical entity remains controversial, with no current specific therapeutic strategies that address diabetes-induced heart failure.

## 4. Evolution of the Disease and Functional Phenotype

The early stages of diabetic cardiomyopathy consist of a hidden, subclinical, asymptomatic period. Prolonged isovolumetric relaxation, impaired early diastolic filling, and increased atrial filling are one of the first manifestations of diabetic cardiomyopathy based on left ventricle hypertrophy and its decreased compliance due to cardiomyocyte stiffness, hypertrophy, and myocardial fibrosis. The underlying mechanisms are increased free fatty acid (FFA), hyperglycemia, insulin resistance, activation of the renin–angiotensin–aldosterone system (RAAS) and the sympathetic nervous system (SNS), calcium pump activity-induced inefficient sequestration of sarcoplasmic reticulum Ca^2+^, oxidative stress, and inflammation [13,16,17].

The second stage is characterized by advanced cardiac remodeling and diastolic dysfunction, with the manifestation of symptoms and signs of heart failure with preserved ejection fraction (HFpEF). Further progression of diabetic cardiomyopathy leads to systolic dysfunction and heart failure with reduced ejection fraction (HFrEF), a dilated left ventricle, shortened ejection period, prolonged pre-ejection performance, and increased filling pressures [13,16]. Contractile and regulatory protein expression abnormalities are involved in the mechanical problems of cardiac contraction seen in this stage [18,19].

## 5. Structural Features of the Diabetic Heart

Left ventricle hypertrophy is a common structural abnormality in diabetic patients [7]. Oxidative stress, insulin resistance, and hyperglycemia promote expression of some cardiomyocyte hypertrophic genes like insulin-like growth factor 1 (IGF-1) receptor, β-myosin heavy chain, or B-type natriuretic peptide [13]. Cardiomyocyte hypertrophy is induced by high insulin levels binding to the IGF-1 receptor [20]. This interaction between IGF-1 and insulin signaling pathways has an important role in hyperglycemia/insulin resistance–induced cardiac hypertrophy and fibrosis in the diabetic heart. Furthermore, concentric left ventricle remodeling in diabetes is also connected to excess cardiomyocyte accumulation of lipids, underlying lipotoxicity as a potential contributor to impaired cardiac performance [21,22]. Another characteristic of the diabetic heart is the development of perivascular and/or interstitial fibrosis as well as coronary microvascular sclerosis and microaneurysms that further promote myocardial stiffness [5,23]. Fibrosis is a result of dysregulation of extracellular matrix degradation and upregulation of transforming growth factor β1 induced by activation of the RAAS and SNS, advanced glycation end products (AGE)–mediated signaling, hyperinsulinemia, and hyperglycemia that induce per se fibroblast and smooth cell proliferation [24,25,26,27]. The diabetic heart is susceptible to both programmed cell death–apoptosis and necrosis as compared to healthy hearts [28]. Hyperglycemia has a proapoptotic effect by inducing oxidative stress and by glycosylation and phosphorylation of p53 and excessive production of angiotensin II [29]. Recently, other mechanisms have been implicated in diabetes such as the dysregulated autophagic response that can also result in excessive cell death [30]. Microvascular abnormalities have been demonstrated in diabetes as a consequence of endothelial dysfunction [31], upregulation of endothelin-1 (ET-1) and downregulation of nitric oxide (NO) [32], autonomic neuropathy [33], which can result in a reduction in coronary blood reserve [14].

## 6. Pathophysiologic Mechanisms Underlying Diabetic Cardiomyopathy

### 6.1. Hyperglycemia

**Figure 1:**
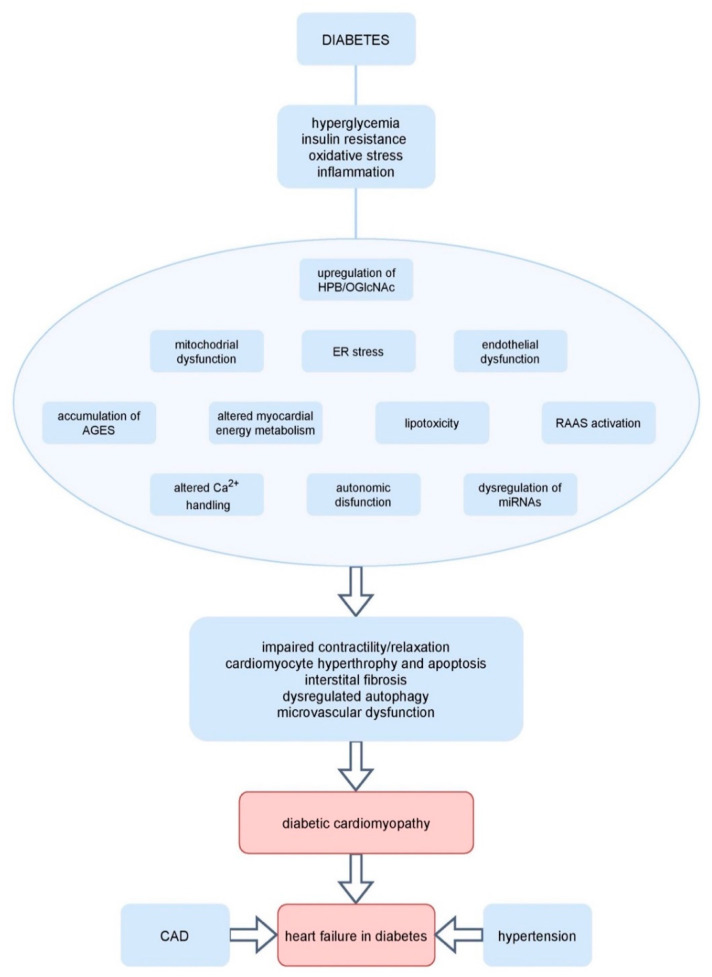
Potential mechanisms involved in diabetic cardiomyopathy. AGE, advanced glycation end products; CAD, coronary artery disease; HBP, hexosamine biosynthesis pathway; miRNA, microRNA; O-GlcNAc, O-linked beta-N-acetylglucosamine; RAAS, renin–angiotensin–aldosterone system; ER endoplasmic reticulum.

Hyperglycemia itself can trigger various maladaptive processes: insulin resistance, hyperinsulinemia, glucose transporter 4 (GLUT4) depletion, accumulation of AGEs, changes in FFA oxidation, altered Ca^2+^ handling, activation of RAAS, generation of reactive oxygen species (ROS), cardiac autonomic neuropathy which will be discussed further in the text (Figure 1) [34,35,36,37,38,39,40].

### 6.2. Insulin Resistance

Insulin resistance has a significant role in the development of cardiovascular disease. The insulin signaling cascade involves various signaling molecules within the cell. Insulin binds to the insulin receptor and stimulates GLUT4 translocation to the cell membrane with subsequent glucose uptake through activation of insulin signaling/docking molecule insulin receptor substrate (IRS)-1/2 and downstream PI3K/protein kinase B (Akt). Moreover, normal cardiac insulin metabolic signaling promotes endothelial nitric oxide synthase activation and bioavailable NO which are responsible for optimal coronary microvascular function [13]. In animal models, abnormalities in heart structure and function are already evident in the prediabetic (insulin-resistance) stage [25]. Cardiac insulin receptor knockout induces mitochondrial dysfunction, decreases cardiac uptake of glucose, increases cardiac ROS generation [41,42]. On the other hand, heart failure itself causes insulin resistance and is a risk factor for the development of type 2 diabetes mellitus making this complex interplay a vicious cycle [43].

### 6.3. Myocardial Energy Metabolism

In contrast to normal heart, there is an increased level of circulating fatty acids in diabetes as insulin fails to suppress hormone-sensitive lipase in adipose tissue and very-low-density lipoprotein secretion in the liver which leads to peroxisome proliferator-activated receptor-α (PPARα) stimulation. The consequence is the upregulation of myocardial fatty acids uptake and metabolism and a decrease in GLUT4 [22]. The increase in fatty acids used in the heart results in a loss of metabolic flexibility, impaired efficiency, abnormal substrate utilization, and decreased energy generation [44,45]. Furthermore, it results in increased production of mitochondrial ROS which leads to reduced bioavailable NO [13]. Excessive cardiomyocyte accumulation of lipids seems to be an important contributor to the development of diabetic cardiomyopathy. Moreover, increased long-chain fatty acyl-CoA concentration is diverted towards the production of lipotoxic intermediates such as diacyl-glycerol and ceramide [21,22,46]. As type 2 diabetes mellitus is linked to decreased ketogenesis as a result of hyperinsulinemia and insulin resistance, ketone bodies could maintain energetic homeostasis in the diabetic heart where there is a reduction in cardiac glucose utilization [47]. Ketones are more efficient than glucose or fatty acids in releasing energy and take part in epigenetic and cellular signaling with antioxidative and anti-inflammatory effects [48,49,50].

### 6.4. Advanced Glycation End Products Modification

AGEs formation is the result of nonenzymatic binding of amine resides on lipids, proteins, or sugar moieties and leads to oxidative stress generation with subsequent thrombogenic, inflammatory, and fibrotic consequences, followed by deterioration of functional and structural integrity of multiple organs [51]. Collagen and elastin, proteins that have slower rates of turnover, are more susceptible to AGE modification, and in this way, AGEs promote vascular and myocardial stiffness with impaired relaxation [52]. The connection with diabetic cardiomyopathy is further strengthened by clinical observation that elevation of AGEs predicted mortality and hospitalization for heart failure in a study of 580 patients with diabetes [53].

### 6.5. Neurohormonal Activation

There is an upregulation of the RAAS, ET-1, and SNS in the diabetic heart which plays an important role in diabetic cardiomyopathy pathogenesis [5]. Inhibition of the aldosterone/mineralocorticoid receptors signaling pathway has been shown to decrease mortality and morbidity among diabetic patients with a mild or moderate degree of heart failure [13]. RAAS activation promotes the proinflammatory M1 phenotype in the myocardium. The abnormalities seen with RAAS activation lead to maladaptive cardiac remodeling with cardiomyocyte apoptosis and hypertrophy, as well as interstitial fibrosis [54,55]. ET-1 is stimulated by hyperglycemia. It is a potent vasoactive and proinflammatory peptide with the ability to stimulate the generation of ROS [56,57]. Cardiac fibrosis and diastolic dysfunction are connected both to reduced NO production and high plasma ET-1 levels in diabetic patients. Furthermore, it was shown that endothelial cell-specific ET-1 knockout was beneficial in preventing diabetic cardiomyopathy and cardiac fibrosis [58]. SNS activation augments β-1 adrenergic receptor signaling which promotes cardiomyocyte apoptosis and hypertrophy, interstitial fibrosis, and impaired cardiac performance [59]. In this regard, cardiac autonomic neuropathy (CAN), a direct consequence of hyperglycemia, implies a reduction of parasympathetic activity with relatively higher SNS activity [34,60]. CAN is associated with abnormal cardiac function in diabetes [61].

### 6.6. Oxidative Stress

Diabetic complications, including cardiac remodeling (namely cardiomyocyte hypertrophy, apoptosis, and cardiac fibrosis), impaired cardiomyocyte calcium handling, endothelial dysfunction, reduced cardiac contractility, and relaxation, are frequently attributed to increased oxidative stress, which arises from the imbalance between the increased generation of ROS and/or a decreased antioxidant defense which is characteristic for diabetes mellitus [62,63,64,65,66]. ROS can directly cause molecular damage (oxidizing DNA, lipid membranes, proteins), activate stress-sensitive pathways in the cells, and induce inflammation [67,68]. Major sources of cardiac ROS derive from uncoupled NO synthases, mitochondrial respiratory chain, and nicotinamide adenine dinucleotide phosphate (NADPH) oxidase which can be triggered by various stimuli such as tumor necrosis factor-α, angiotensin II and ET-1. Other minor sources of cardiac ROS include xanthine oxidase, cyclooxygenase, myeloperoxidase, and lipoxygenase. In the hyperglycemic state, endogenous antioxidants such as superoxide dismutase, glutathione peroxidase, and catalase, thioredoxin are all reduced [5,69].

### 6.7. Inflammation

Inflammation significantly contributes to the development of diabetic cardiomyopathy [70]. Proinflammatory responses seen in diabetes occur in various types of cardiac cells, including fibroblasts, cardiomyocytes, coronary smooth muscle, and endothelial cells [13]. For instance, proinflammatory M1 macrophage polarization is enhanced, whereas macrophage M2 anti-inflammatory response is reduced in diabetic myocardium [16]. NLRP3 (NOD-, LRR-, and pyrin domain-containing protein 3) is an intracellular sensor, that can result in activation of NLRP3 inflammasome due to diverse stimuli such as hyperglycemia and ROS and then leading to caspase-1 activation. Activated caspase-1 cleaves interleukin-18 and interleukin-1β precursors and promotes various inflammatory pathways involving chemokines, nuclear factor κ-light-chain-enhancer of activated B cells (NF-κB), or ROS [71,72]. Inflammation in the heart is implicated in systolic dysfunction, diastolic stiffness, and increased fibrosis [73,74]. Furthermore, heart failure and inflammation mutually enhance each other producing a vicious circle [75].

### 6.8. Mitochondrial Dysfunction

In type 2 diabetes, to product adenosine triphosphate (ATP), mitochondria switch from glucose to FFA oxidation which is accompanied by impaired oxidative phosphorylation, elevated mitochondrial ROS generation, and reduced efficiency in energy production [14,76]. Moreover, mitochondrial dysfunction increases Ca2+ overload-induced opening of the mitochondrial permeability transition pores leading to cardiomyocyte necrosis and autophagy [77]. Mitochondrial ROS are now appreciated to function as signaling molecules. It has been postulated that they are generally induced by cell stress and serve as an alarm to notify the cell that the extracellular environment is changing. A stressor that is not compatible with cell viability produces larger quantities of ROS, that induce cell damage and subsequent cell death. In the context of diabetes, there is a connection between aberrant ROS formation and oxidative stress with the development of diabetic cardiac abnormalities [78,79].

### 6.9. Altered Ca^2+^ Handling

During cardiac excitation–contraction coupling, after depolarization of the sarcolemma, Ca^2+^ enters the cytoplasm through voltage-sensitive L-type Ca^2+^ channels which trigger Ca^2+^ release from the sarcoplasmic reticulum (SR) through ryanodine receptors and then bind troponin C to provoke contraction. Cardiac relaxation initiates when Ca^2+^ is transported back into the SR by an ATP-dependent calcium pump (SERCA, sarco-endoplasmic reticulum calcium-ATPase), and the remaining Ca^2+^ is pumped out by the sarcolemma Na^1+^/Ca^2+^ exchanger and the Ca^2+^ pump in the plasma membrane. [80,81,82]. There is a disturbance of Ca^2+^ regulation in diabetic heart–impaired Ca^2+^ handling by all these regulators prolong action potential duration and diastolic relaxation time [16]. Elevated basal intracellular Ca^2+^, prolongation of its decay, slowed transients, SERCA dysfunction, have been documented in the hearts of type 2 diabetic mice and similarly in type 1 diabetic rodent models [83,84,85,86]. Dysfunctional cardiomyocyte Ca^2+^ handling seems to play a key role in the development of the early diastolic impairment in diabetic cardiomyopathy.

### 6.10. O-Linked Beta-N-acetylglucosamine (O-GlcNAc) Protein Modification

In normal conditions, the hexosamine biosynthesis pathway (HBP) diverts a small amount of fructose-6-phosphate metabolism from glycolysis to generate the O-GlcNAc sugar moiety that can further modify proteins. Transient activation of O-GlcNAc signaling is cytoprotective but its sustained elevation that is found in diabetes is involved in impaired mitochondrial function, abnormal insulin metabolic signaling, endothelial dysfunction, myocardial excitation–contraction coupling, and cardiomyocyte apoptosis [5,87,88,89,90,91]. Taken together, HBP came out as a contributor to the development of diabetic cardiomyopathy.

### 6.11. microRNAs (miRNAs) Dysregulation

Studies in cell cultures, animal models, and clinical studies have associated diabetic cardiomyopathy with increased or decreased expression of miRNAs—a group of non-coding RNA molecules that control the expression of transcriptional and post-transcriptional target genes. miRNAs expression experiences alterations in the diabetic heart. Furthermore, they regulate various processes that are involved in diabetic cardiomyopathy pathogenesis such as fibrosis, apoptosis, autophagy, Ca2+ handling, mitochondrial function, and ROS production [92,93]. Nevertheless, miRNA dysregulation partially overlaps with heart failure miRNA pattern, so more investigations are needed to determine a specific miRNA expression characteristic for diabetic cardiomyopathy [5].

### 6.12. Endoplasmic Reticulum Stress

Lipotoxicity, ROS, inflammation, accumulation of misfolded proteins induce endoplasmic reticulum stress, leading to unfolded protein response which ultimately augments cell autophagy through a Ca^2+^-dependent pathway and apoptosis, one of the key risk factors for the development of diabetic cardiomyopathy [16,94,95].

### 6.13. Epicardial Adipose Tissue

Epicardial fat, physically next to the myocardium, shares the same microcirculation with the myocardium. It can be protective regarding myocardial stress and inflammation, but in diabetes it becomes dysfunctional, and subjected to the maladaptive adipocyte biology characterized by hypertrophy, increased inflammation, and lipolysis, promoting cardiovascular injuries and myocardial steatosis [96].

## 7. Signaling Pathways Contributing to Diabetic Cardiomyopathy

### 7.1. AMP-Activated Protein Kinase (AMPK)

Regulates various cellular processes, such as cell growth, energy metabolism, autophagy, and apoptosis. In the heart, AMPK is responsible for the activation of glucose uptake, glycolysis, and FFA oxidation in the condition of cellular stress. It has been shown that AMPK can also restore autophagy, reduce cardiac apoptosis, prevent and ameliorate diabetic cardiomyopathy [97].

### 7.2. Variations in Activations of Cardiac Peroxisome Proliferator-Activated Receptors (PPARs)

PPARs (α, β/δ, and γ) belong to the nuclear receptor superfamily of transcription factors and are expressed in the heart, playing a pivotal role in myocardial lipid and glucose metabolism. They are also involved in oxidative stress and inflammation [13]. PPARα is relatively highly expressed in cardiomyocytes. In transgenic mice, cardiac PPARα overexpression causes decreased SR uptake of Ca^2+^, increased atrial and B-type natriuretic peptide expression, left ventricle hypertrophy, and systolic dysfunction [22]. Deletion of cardiac PPAR-α, on the other side, has been shown to induce reduced cardiac FFA oxidation. The altered fatty acid-metabolizing proteins expression in PPARα^−/−^ is associated with myocardial damage and fibrosis [98]. In rodent cardiomyocytes, chronic exposure to elevated FFAs reduces the expression of PPAR-α which inhibits FFA oxidation and increases intracellular fat accumulation reducing heart function [99,100]. Like PPAR-α, PPAR-β/δ is abundantly expressed in the heart playing a different role in myocardial metabolism since its overexpression enhances the capacity for myocardial glucose utilization and increases glycolytic gene expression. Furthermore, PPAR-β/δ transgenic mice did not accumulate myocardial lipid and had a normal cardiac function which identifies PPAR-β/δ as a possible target metabolic modulation therapy for cardiac dysfunction in diabetes [5,101]. PPAR-γ has relatively low expression in the heart but has antihypertrophic and anti-inflammatory myocardial effects since its agonists, which are approved for the treatment of type 2 diabetes, improve cardiomyocyte insulin sensitivity and glucose uptake making them possibly beneficial for cardiac function [5,13].

### 7.3. Cardiac Mitogen-Activated Protein Kinase (MAPK) Signaling

MAPKs are serine/threonine-specific protein kinases whose activation plays a role in the pathogenesis of diabetic cardiomyopathy regulating cardiac growth, hypertrophy, and remodeling. There are 4 important MAPK subfamilies including ERK1/2, p38 MAPK, JNKs, and ERK5 [5,102]. p38 MAPK (α, β, γ, δ) are stress-activated kinases associated with a wide spectrum of cardiac pathologies, mostly p38α MAPK and p38γ (preferentially expressed in cardiac muscle) since expression of p38β MAPK and p38δ MAPK is limited in the myocardium. Hyperglycemia, as well as oxidants and pro-inflammatory cytokines commonly elevated in diabetes, stimulate the p38 MAPK signaling pathway contributing to myocyte death, cardiac dysfunction, and fibrosis as a response to ischemia [5,103]. Even though the functional role of *extracellular signal-regulated kinase 1/2* (ERK 1/2) is not fully clear in diabetic hearts, some studies showed increased phosphorylation of ERK in diabetic mice after STZ induction of diabetes [5,104]. *c-Jun N-terminal kinases* (JNKs) activation can be caused by inflammatory cytokines and oxidative stress in high glucose states leading to increased cardiomyocyte apoptosis, so both MAPK and JNK signaling activations seem to contribute to diabetic cardiomyopathy development [13].

### 7.4. Protein Kinase C (PKC) Activation

PKC activation is triggered by hyperglycemia and insulin resistance and further promoted by oxidative stress, inflammation, and increased RAAS and SNS activity. PKC overexpression, especially PKC β2 isoform, in diabetic myocardium induces cardiomyocyte hypertrophy and diastolic dysfunction by altering caveolin-3 expression and insulin metabolic Akt/eNOS signaling [13,105].

### 7.5. NF-κB Activation

NF-kB is one of the key transcription factors involved in the expression of proinflammatory cytokines, profibrotic and hypertrophy-related genes, and cell survival, thus NF-kB activation plays an important role in diabetic cardiomyopathy genesis. Its activation in the diabetic heart causes proinflammatory cytokines release (interleukins, TNFα), reduces the bioavailability of NO, and amplifies oxidative stress resulting in endothelial dysfunction in T2D [13,106].

### 7.6. Transcription Factor Cyclic Adenosine 5-Monophosphate-Responsive Element Modulator (CREM) Activation

CREM is a transcription factor involved in the control of cAMP signaling and cardiotoxicity-associated gene expression that may lead to cardiac fibrosis by altering the genetic landscape of cardiac proteins. Both hyperglycemia and elevated free fatty acids induce CREM overexpression causing pancreatic B cell dysfunction by inhibiting insulin gene transcription and insulin secretion [13,107,108].

### 7.7. Exosomes Abnormalities

Exosomes are vesicles in the extracellular fluid that are important for cell-to-cell communication. In the state of glucose deprivation, cardiomyocytes increase the synthesis and secretion of exosomes transporting glycolytic enzymes and glucose transporters directly to endothelial cells facilitating glucose uptake [109]. Some data explain the antiangiogenic function of cardiomyocytes by transferring miR-320 loaded exosomes to endothelial cells which may be part of the underlying mechanism of myocardial ischemia in diabetes [110].

### 7.8. Abnormalities of Nuclear Factor Erythroid 2–Related Factor 2 (Nrf2)

Nrf2 plays an important role in the regulation of insulin sensitivity in the heart. Depressed expression of cardiac Nrf2 is associated with ERK activation that mediates oxidative stress-induced insulin resistance and downregulation of glucose metabolism, while Nrf2 activation suppresses ERK activity and reverses oxidative stress-induced insulin resistance [111].

### 7.9. SGLT2 Abnormalities

A family of active glucose transporter proteins-the sodium-dependent glucose cotransporters (SGLTs) consists of SGLT1 which are widely expressed in numerous organs and SGLT2 that is mainly expressed in the renal proximal tubule. Glucose is normally filtered into the urine at the glomerulus and then reabsorbed in the proximal tubule by SGLT2 (90% of filtered glucose) and SGLT1 (the remaining 10%). In a high glucose state SGLT2 expression is increased, paradoxically resulting in the elevated threshold for urinary glucose excretion. SGLT2 inhibitors inhibit glucose and sodium reabsorption from the renal proximal tubules and thus promote urinary glucose excretion, whereas other hypoglycemic drugs are mainly focused on stimulating glucose utilization, such as increasing insulin sensitivity, tissue glucose uptake, and restoring β-cell activity [112,113,114,115]. The major cardiovascular outcome trials regarding SGLT2 inhibitors showed that cardiovascular risk reduction seen in these studies was mostly driven by a substantial reduction in heart failure risk, which was unexpected (Table 1) [10,11,12]. Moreover, recently, impressing results came from the DAPA-HF trial in patients with heart failure and without diabetes where dapagliflozin reduced heart failure risk for HFrEF patients regardless of diabetes status [116]. As their cardiovascular benefits were shown very early in the mentioned trials (after several weeks), the glucose-lowering and antiatherosclerotic-mediated effects became less important and multiple other mechanisms, direct cardiac and systemic, are suggested to explain SGLT2 inhibitors mechanisms of action [117]. Some of them are renoprotective [118] and hemodynamic effects [119], anti-inflammatory, antioxidative [112], antifibrotic [120], and antiapoptotic properties, reduction of uric acid levels [121] and epicardial adipose tissue [122], attenuation of glucotoxicity [123], sodium-hydrogen exchange inhibition [124,125], modification of neurohumoral system [126,127,128], and cardiac fuel energetics [48]. After their benefits have been translated to non-diabetic patients, the situation regarding SGLT2 inhibitors has become even more complicated. Furthermore, one question arises–whether we are missing something in the pathophysiology of heart failure?

### 7.10. Mammalian Target of Rapamycin (mTOR) Signaling

mTOR is a serine/threonine-protein kinase that belongs to a PI3K-related family member. It mainly exists in two forms: mTORC1, which regulates cell proliferation, metabolic reactions, and mTORC2 [129]. Enhanced mTOR may serve as a convergence point for abnormalities involving the interplay between autophagy and endoplasmic reticulum stress in the diabetic heart [13]. Diabetic hearts exhibit activation of the mTOR signaling pathway, and it has been shown that activation of AMPK by metformin inhibits the mTOR pathway and restores cardiac autophagy in mice [130].

## 8. Clinical Diagnosis

The most reasonable approach to identify diabetic cardiomyopathy is the detection of structural and functional changes in the left ventricle with the exclusion of other entities contributing to heart failure [14]. Myocardial structure and function changes could be detected before the appearance of heart failure symptoms [62]. Structural changes (left ventricle hypertrophy, interstitial fibrosis) could be identified by invasive (endomyocardial biopsy) and non-invasive techniques (echocardiography, magnetic resonance imaging, multislice computed tomography) [131]. Functional changes (diastolic and systolic dysfunction and limited diastolic or/and systolic functional reserve) are identified by means of echocardiography (especially tissue Doppler imaging, pulsed wave Doppler, 2D-speckle-tracking echocardiography, exercise echocardiography) [14]. Characterization of metabolic changes in the diabetic heart is possible by magnetic resonance spectroscopy (^1^H-MRS to detect an increase in myocardial triglyceride content and ^31^P-MRS), as well as by single-photon emission computed tomography and positron emission tomography that can show alterations in myocardial glucose utilization and fatty acid metabolism. Nuclear imaging provides information regarding diabetic autonomic neuropathy as well [132,133,134,135].

To prevent the initiation and progression of diabetic cardiomyopathy, a sensitive method of diagnosing the presence of this entity is crucial. Still, clinical diagnostic methods are not well defined [136]. Recently, 2D-speckle-tracking echocardiography (STE) has been validated (against cardiac magnetic resonance, tissue Doppler imaging, sonomicrometry) in numerous patients, including those with diabetes. It has been shown that 30–50% of patients with type 2 diabetes and normal 2D ejection fraction have an abnormal global longitudinal strain measured by STE. Consequently, it seems reasonable that STE could be used for the early detection of subclinical cardiac dysfunction among diabetic patients [137]. Also, STE could be used for observing different stages of diabetic cardiomyopathy detecting relative changes from baseline rather than an absolute cut-off value as in cardio-oncology. Nevertheless, larger studies with echocardiographic evaluation and pathway-specific biomarkers that could be validated against hard endpoints are needed [62].

## 9. Therapeutic Possibilities

Despite the growing interest in diabetic cardiomyopathy, there are no specific guidelines for the treatment of this entity in clinical practice. Here are listed some of the possibilities for pharmacotherapy in heart failure and diabetes mellitus, with their potential consideration and limitation in everyday practice.

### 9.1. Heart Failure Prevention in Diabetes

#### 9.1.1. Lifestyle Interventions

The moderate-intense activity could help control insulin resistance, inflammation, and visceral adiposity that are all related to the heart in the setting of diabetes [62]. Beneficial effects of exercise on the diabetic heart have been also shown in animal studies, where exercise-trained rodent models of diabetes have demonstrated improved outcomes associated with attenuation of collagen deposition, better calcium homeostasis, and presence of more intact mitochondria [138,139,140].

#### 9.1.2. Metabolic Therapies

HbA1c as a marker of glycemic control has been associated with risk for heart failure, with a 16% fall of heart failure for each 1% decrement of HbA1c and an 8% increment per 1% increase of this marker [141]. With better glycemic control, the glycation of lipids and proteins that have effects on the myocyte as well as insulin resistance should be reduced. Glucose-lowering pharmacotherapy in patients with diabetes is discussed further in the text.

#### 9.1.3. Targeting Oxidative Stress

Superoxide dismutase-, catalase, and glutathione peroxidase-, thioredoxin-based approaches have all shown beneficial effects in various experimental models of diabetic cardiac pathologies [142,143,144,145,146]. Antioxidant approaches, such as pharmacological inhibition of nicotinamide adenine dinucleotide phosphate oxidase and MitoQ and coenzyme Q10 supplementation are promising strategies for managing cardiac complications induced by diabetes, as has been suggested from early-stage clinical and preclinical studies [147,148,149,150].

#### 9.1.4. SGLT2 Inhibitors

Interestingly, this class of drugs has demonstrated robust benefits in reducing heart failure hospitalization regardless of a history of heart failure or existing atherosclerotic disease, which make them potential drugs for primary prevention in heart failure [151].

#### 9.1.5. RAAS Inhibition

Inappropriate activation of RAAS can negatively influence cardiac insulin metabolic signaling [54]. Angiotensin-converting-enzyme inhibitors (ACEi) have the potential to improve insulin sensitivity and resistance, they exert beneficial effects on microcirculation and prevent diabetic cardiomyopathy in preclinical models of both type 1 and 2 diabetes [148,152,153,154].

### 9.2. Conventional Therapies for Diabetes

There is a paucity of specific data to guide HbA1c goals in patients with heart failure and diabetes, so it is suggested that a target range of HbA1c is between 7% and 8%. This is consistent with guidelines for patients with diabetes and serious comorbidities [9]. Glucose-lowering agents in patients with diabetes mellitus type 2 are:

#### 9.2.1. Metformin

The preferred drug for diabetes mellitus type 2 pharmacotherapy initiation in the absence of contraindications [155]. It was previously contraindicated in heart failure due to the risk of lactic acidosis [9]. But, in one meta-analysis of nearly 34,000 patients, metformin was associated with reduced mortality in patients with heart failure compared with controls [156]. Furthermore, it has been shown that metformin is associated with improved cardiac function and alleviation of apoptosis in diabetic mice, as well as protection of cultured cardiomyocytes from cell death during exposure to H_2_O_2_ via AMPK activation [157,158].

#### 9.2.2. Sulfonylurea Agents

There are no randomized trials examining the effects of sulfonylurea drugs on clinical outcomes in diabetes. Based on the available data, the use of other agents, such as SGLT2 inhibitors and metformin, is preferable. In various observational studies seemed that sulfonylurea therapy may be related to increased risk of heart failure events compared with dipeptidyl peptidase-4 (DPP-4) inhibitors or metformin [159,160].

#### 9.2.3. Insulin

Some observational studies indicated an increase in heart failure with insulin use [8,161]. Nevertheless, it is sometimes required to achieve glycemic control, but caution is required since it is associated with the risk of hypoglycemia and weight gain.

#### 9.2.4. Thiazolidinediones

These drugs are associated with increased heart failure events [162,163,164]. It seems that volume expansion caused by raised sodium reabsorption in the kidney is the predominant mechanism for its adverse events [165].

#### 9.2.5. Incretin-Based Therapies

Glucagon-like peptide-1 (GLP-1) receptor agonists have shown mostly beneficial effects on cardiovascular outcomes in large-scale post-marketing cardiovascular outcomes trials. On the other side, they were not associated with heart failure hospitalization reduction. Furthermore, there are conflicting results regarding their potential benefits in heart failure in animal and human studies [166,167,168,169]. Clinical outcomes with DPP-4 inhibitors showed no or partially negative effects on heart failure hospitalization, despite data from animal models and several smaller studies that have suggested potential beneficial effects of these agents [170]. The possible role of different incretin-based therapies in patients with diabetic cardiomyopathy and heart failure is not yet fully determined, and caution is needed when translating mechanisms derived from animal data.

#### 9.2.6. SGLT2 Inhibitors

The first class of glucose-lowering drugs that have demonstrated a consistent and robust reduction in the risk of heart failure hospitalization in patients with diabetes make them game-changers in cardiometabolic pharmacotherapy. Additionally, the efficacy in primary and secondary prevention of heart failure has already translated to efficacy in the treatment of heart failure in HFrEF patients with or without diabetes, but they may also be of value in the treatment of HFpEF [171,172]. The potential mechanisms of their action have already been mentioned before. Furthermore, it has been shown that vasoactive agents, such as atrial natriuretic peptide-ANP and ET-3 inhibit SGLT2 activity in the kidney. In the case of SGLT2 transporters inhibition, ANP could exert other functions other than diuretic and natriuretic activity, e.g., RAAS inhibition, blunting sympathetic response, and protection against angiotensin II-induced cardiac remodeling minimizing macrophage infiltration and expression of pro-inflammatory mediators. In this way, SGLT2 inhibitors could synergistically work with angiotensin receptor neprilysin inhibitors (ARNIs). In theory, this would be of value especially in the failing heart when the natriuretic peptides are markedly raised [172,173,174].

### 9.3. Conventional Therapies for Heart Failure

The therapy for established HFrEF is the same, whether or not the patient has diabetes: RAAS inhibitors, ARNIs, β-blockers, ivabradine, implantable cardioverter defibrillators/cardiac resynchronization therapy in HFrEF regardless of diabetes status given the strength of the data regarding benefits of these drugs/devices. No therapy has been shown to reduce mortality and morbidity in HFpEF yet. The non-cardiovascular and cardiovascular comorbidities are often associated with different phenotypes of HFpEF so they should be managed appropriately with interventions that improve symptoms without exacerbating heart failure [175,176].

### 9.4. Metabolic Modulators

The potential target of therapeutic agents to improve cardiac function is substrate metabolism in the heart.

#### 9.4.1. Trimetazidine

This agent has been shown to ameliorate diabetic cardiomyopathy features in animal models of diabetic cardiomyopathy and reverse insulin resistance [177]. Trimetazidine is an anti-ischemic and antioxidant agent that modifies energy metabolism by partial inhibition of fatty acid oxidation and increased glucose oxidation. It ameliorates the prognosis of diabetic patients with ischemic heart disease, but whether it can prevent the development of heart failure in diabetic patients has not been investigated in clinical studies [7].

#### 9.4.2. Perhexiline

It shifts metabolism towards glucose and lactate utilization through the inhibition of carnitine palmitoyltransferase. It has been linked to improvements in left ventricular systolic function, skeletal muscle energetics, and peak exercise oxygen consumption. Furthermore, it ameliorates symptoms in patients with chronic heart failure [7,178].

#### 9.4.3. Meldonium

Reduces L-carnitine biosynthesis and uptake shifting metabolism towards glucose consumption. In obesity and impaired glucose tolerance animal models, it decreased insulin levels and increased cardiac and hepatic PPAR-α activity [179].

#### 9.4.4. Fenofibrate

With activating PPAR-α in specific circumstances such as diabetes, skeletal muscles may use the substrate that is available offering two advantages: the muscles’ metabolic needs are continuously underpinned and fewer potentially harmful lipid byproducts are accumulated in the diabetic heart [180]. PPAR-α agonists, once again, underline the lack of metabolic flexibility characteristic for the diabetic heart.

#### 9.4.5. GLP-1 Agonists and Mineralocorticoid Receptor Blockers

These agents are implicated in the modification of myocardial steatosis reversing the concentric left ventricle remodeling process [167,181].

#### 9.4.6. SGLT2 Inhibitors

These pleiotropic agents could also reduce epicardial adipose tissue volume, a form of visceral fat that has no anatomical barriers with the myocardium and that is implicated in the pathogenesis of diabetic heart disease through altered paracrine regulation of cytokines and adipokines [7,122,182]. Since SGLT2 inhibitors can redirect metabolism from glucose to fatty acid oxidation which augments the synthesis of ketones, these agents could be used to induce alternate fuel sources in the failing heart [48].

### 9.5. Potential Therapies Targeting Signaling Pathways

#### 9.5.1. p38 MAPK Inhibition

Pharmacological inhibition of p38 MAPK decreases cardiac inflammation and systolic dysfunction as showed in streptozotocin-induced diabetic mice treated with p38 MAPK inhibitor which targets p38 kinases as a promising therapeutic possibility in the prevention of diabetic cardiomyopathy or its progression [73].

#### 9.5.2. PKC β2 Inhibition

Inhibition of PKC β2 activation improves cardiac diastolic function by recovering caveolin-3 expression and therefore may represent a possible treatment option for diabetic cardiomyopathy [13,105]. The efficacy of ruboxistaurin as a PCK β inhibitor in the treatment of diabetic cardiomyopathy is being studied in clinical trials [5].

#### 9.5.3. Phosphodiesterase Type 5 Inhibition

Sildenafil as an inhibitor of phosphodiesterase type 5 may prevent some of the hyperglycemia-induced changes in cardiomyocyte gene expression counteracted the increase of CREM [107].

#### 9.5.4. AMPK Activation

AMPK activation has a favorable part in preventing diabetic cardiomyopathy progression. In a study by Xie et al., chronic metformin treatment activated AMPK, inhibited cardiomyocyte apoptosis, and restore autophagic activity in diabetic heart tissue [183].

#### 9.5.5. ET-1 Targeting

Targeting endothelial cell-specific ET-1 could be effective in the treatment of diabetic cardiomyopathy [58].

#### 9.5.6. HBP and O-GlcNAc Targeting

HBP and O-GlcNAc as potential therapeutic targets could also be a potential strategy in the therapy of diabetic cardiomyopathy [184].

#### 9.5.7. NF-κB Inhibition

Pyrrolidine dithiocarbamate restores cardiac function in an animal model of type 2 diabetes inhibiting NF-κB and subsequently improving mitochondrial structural integrity, inhibiting oxidative stress, and increasing ATP synthesis [185].

#### 9.5.8. Nrf2 Activity

Restoring of Nrf2 activity prevents inflammation, lipid accumulation, and fibrosis induced by diabetes, providing another potential therapeutic target in diabetic cardiomyopathy [186].

#### 9.5.9. Heat Shock Protein20-Engineered Exosomes

Heat shock protein20-engineered exosomes might regulate cardiomyocyte exosome secretion and, in this way, restore the hyperglycemia-induced cardiac dysfunction in diabetic animal models [187].

#### 9.5.10. microRNAs

microRNAs could also be a potential therapeutic target in diabetic cardiomyopathy. According to Katare et al., transfection with anti-miR1 promotes prosurvival signals in cardiac progenitor cells and cardiomyocytes exposed to high-level glucose [188].

### 9.6. Correction of Intestinal Dysbiosis

Various studies have shown that diabetic cardiomyopathy is associated with altered intestinal microbiota and changes in the synthesis of bacterial metabolites that could have direct deleterious effects on cardiac contractility [189]. It has been shown that a decrease in the number of bacteria producing butyrate could be linked with both the presence of diabetes and cardiac insufficiency [190]. Nevertheless, more randomized clinical trials are needed to confirm whether changes to the microbiota can improve cardiac insufficiency.

## 10. Gene Therapy

It is possible to modulate the expression (up or down-regulation) of specific cardiac genes for the treatment of diabetic cardiomyopathy. Systemic administration of proviral integration site for Moloney murine leukemia virus-1 (PIM-1) via cardiotropic serotype-9 adeno-associated virus (AAV) increased Pim-1 expression and prevented cardiac apoptosis, fibrosis, and heart failure in an animal diabetic model [188]. Gene delivery of cardiac phosphoinositide 3-kinase (caPI3K) using AAV6 improves left ventricle systolic function in mice with left ventricle hypertrophy and prevents streptozotocin-induced diabetic cardiomyopathy [5,7]. We are expecting the translation of gene therapies into clinical practice.

## 11. Cardiomyopathy in Type 1 and Type 2 Diabetes Mellitus

Cardiomyopathies in type 1 and type 2 diabetes mellitus share common pathophysiological mechanisms, such as hyperglycemia, lipotoxicity, glucotoxicity, inflammation, and oxidative stress which are the principal mechanisms in the development of diabetic cardiomyopathy, even though the etiologies of type 1 and type 2 diabetes mellitus differ [191]. It is also known that the most prevalent complications of both type 1 and type 2 diabetes are cardiovascular diseases, retinopathy, nephropathy, and neuropathy. Furthermore, it is very intriguing that metabolic disturbances could produce a ‘metabolic memory’, that can initiate multiple epigenetic mechanisms involved in the evolution of diabetic cardiomyopathy, regardless of whether glucose levels have normalized [192,193,194]. However, in type 2 diabetes mellitus associated cardiomyopathy, cardiomyocyte hypertrophy and concentric left ventricular remodeling which increase ventricular stiffness and promote diastolic dysfunction are more prominent resulting in HFpEF early in the disease progression while HFrEF occurs later. On the other hand, the characteristics of cardiomyopathy associated with type 1 diabetes are cardiomyocyte loss, left ventricle remodeling, and increased myocardial collagen deposition, which impairs systolic function early in the course of the disease [191].

## 12. Conclusions

Diabetic cardiomyopathy is a challenging diagnosis, made after excluding other potential entities, treated with different pharmacotherapeutic agents targeting various pathophysiological pathways that we poorly understand. Therefore, it is crucial to make more efforts in unraveling the mystery behind this distinct cardiomyopathy that accompanies closely diabetes, a disease with pandemic proportions. A favorable treatment agent would be the one that has pleiotropic effects influencing multiple mechanisms implicated in the evolution of diabetic cardiomyopathy and, in this way, reducing the need for polypragmasia. Furthermore, investigations are needed in translating the preclinical results in everyday practice as well as to clarify potential enhancing benefits of various combination therapy. As a systemic disease, diabetes also affects right ventricular function and structure, which needs to be further investigated and determined. Without a doubt, there is still a lot of work to do, but the results could be of great clinical importance.

## Figures and Tables

**Table 1:** Major Cardiovascular Outcome Trials with SGLT2 inhibitors.

PARAMETERS	CANVAS Program	DECLARE-TIMI 58	EMPA-REG OUTCOME
Intervention	Canagliflozin/placebo	Dapagliflozin/placebo	Empagliflozin/placebo
Median follow-up(years)	3.6	4.2	3.1
Number of patients	10142	17160	7020
Prior cardiovascular disease/heart failure (%)	65.6/14.4	40/10	99/10
Primary outcome (3-point MACE)	0.86 (95% CI 0.75–0.97) Noninferiority, *p* < 0.001 Superiority, *p* = 0.02	0.93 (95%CI 0.84–1.03) Noninferiority, *p* < 0.001 Superiority, *p* = 0.17	0.86 (95% CI 0.74–0.99) Noninferiority, *p* < 0.001 Superiority, *p* = 0.04
Cardiovascular death	0.87 (0.72–1.06)	0.98 (0.81–1.17)	0.62 (0.49–0.77) *
Myocardial infarction	0.89 (0.73–1.09)	0.89 (0.77–1.01)	0.87 (0.70–1.09)
Stroke	0.87 (0.69–1.09)	1.01 (0.84–1.21)	1.18 (0.89–1.56)
Heart failure hospitalization	0.67 (0.52–0.87) *	0.73 (0.61–0.88) *	0.65 (0.50–0.85) *
All-cause mortality	0.87 (0.74–1.01)	0.93 (0.82–1.04)	0.68 (0.57–0.82) *

MACE, major cardiac adverse event; * significant.
